# Analysis of Protein Oxidative Modifications in Follicular Fluid from Fertile Women: Natural Versus Stimulated Cycles

**DOI:** 10.3390/antiox7120176

**Published:** 2018-11-27

**Authors:** Irantzu Pérez-Ruiz, Susana Meijide, María-Luisa Hérnandez, Rosaura Navarro, Zaloa Larreategui, Marcos Ferrando, María-Begoña Ruiz-Larrea, José-Ignacio Ruiz-Sanz

**Affiliations:** 1 Free Radicals and Oxidative Stress (FROS) research group of the Department of Physiology, Medicine and Nursing School, University of the Basque Country UPV/EHU, 48940 Leioa, Spain; irantzu.perez@ehu.eus (I.P.-R.); sumeijide@gmail.com (S.M.); luisa.hernandez@ehu.eus (M.-L.H.); rosaura.navarro@ehu.eus (R.N.); joseignacio.ruizs@ehu.eus (J.-I.R.-S.); 2 BioCruces Health Research Institute, Plaza de Cruces s/n, 48903 Barakaldo, Spain; 3 Valencian Institute of Infertility (IVI-RMA)-Bilbao, 48940 Leioa, Spain; zaloa.larreategui@ivirma.com (Z.L.); marcos.ferrando@ivirma.com (M.F.)

**Keywords:** assisted reproduction, follicular fluid, oxidative stress, aminoadipic semialdehyde, glutamic semialdehyde, gas chromatography-mass spectrometry, female infertility

## Abstract

Oxidative stress is associated with obstetric complications during ovarian hyperstimulation in women undergoing in vitro fertilization. The follicular fluid contains high levels of proteins, which are the main targets of free radicals. The aim of this work was to determine specific biomarkers of non-enzymatic oxidative modifications of proteins from follicular fluid in vivo, and the effect of ovarian stimulation with gonadotropins on these biomarkers. For this purpose, 27 fertile women underwent both a natural and a stimulated cycle. The biomarkers, glutamic semialdehyde (GSA), aminoadipic semialdehyde (AASA), *N^ε^*-(carboxymethyl)lysine (CML), and *N^ε^*-(carboxyethyl)lysine (CEL), were measured by gas-liquid chromatography coupled to mass spectrometry. Results showed that follicular fluid contained products of protein modifications by direct metal-catalyzed oxidation (GSA and AASA), glycoxidation (CML and CEL), and lipoxidation (CML). GSA was the most abundant biomarker (91.5%). The levels of CML amounted to 6% of the total lesions and were higher than AASA (1.3%) and CEL (1.2%). In the natural cycle, CEL was significantly lower (*p* < 0.05) than in the stimulated cycle, suggesting that natural cycles are more protected against protein glycoxidation. These findings are the basis for further research to elucidate the possible relevance of this follicular biomarker of advanced glycation end product in fertility programs.

## 1. Introduction

Free radicals are highly reactive molecular species that are inherent to aerobic life. Proteins are one of the major targets of free radical attack, not only because of their high concentration in all compartments but also because some amino acids (tyrosine, methionine, arginine, proline, and lysine) are prone to non-enzymatic modifications [1]. Proteins are involved in practically all physiological processes, and their oxidative modifications may have detrimental effects in an organism [2,3]. A hallmark of oxidative protein damage is the introduction of carbonyl groups into amino acid residues. Carbonyl groups in proteins may directly arise by metal-catalyzed oxidation of specific amino acids. Alternatively, during the protein glycation and the peroxidation of polyunsaturated fatty acids, reactive aldehydes (methylglyoxal, glyoxal, and 4-hydroxynonenal) are formed. These compounds react with residues of lysine in proteins generating covalent adducts, thus contributing to total carbonylation. Therefore, the quantification of total carbonyl groups in proteins constitutes a global marker of protein oxidation independent of the radical initiator [4,5].

Multiple residues in proteins are susceptible to non-enzymatic modifications, which presents a challenge in undertaking the analysis, but also the possibility of getting a fingerprint. Thus, the metal-catalyzed oxidation renders glutamic semialdehyde (GSA) and aminoadipic semialdehyde (AASA) as main carbonyl products [6]. Lysine residues in proteins are targets for specific covalent addition of reactive compounds derived from glucose metabolism [7] and lipid peroxidation [8]. As a consequence, the *N^ε^*-(carboxymethyl)lysine (CML) and *N^ε^*-(carboxyethyl)lysine (CEL) advanced glycation end products (AGEs) are formed, the last one arising specifically from non-enzymatic decomposition of glycolytic intermediates [9]. All these compounds represent specific indices of in vivo oxidative modifications of amino acids in proteins.

Controlled ovarian stimulation with exogenous gonadotropins is generally used in assisted reproduction in order to recover a synchronous cohort of mature oocytes and thus improve the results of in vitro fertilization. However, the treatment involves clinical problems, such as pregnancy and perinatal complications [10,11], multiple pregnancies [12], and ovarian hyperstimulation syndrome [13]. Ovarian hyperstimulation also causes deterioration of the quality and maturity of oocytes in some patients, including patients with polycystic ovary syndrome (PCOS) and unexplained poor responders [14]. The follicular fluid is the microenvironment for the developing oocyte and is enriched in proteins. A large number of proteins have been identified in this fluid by proteomic analysis [15,16,17]. More than 60% of the intrafollicular proteins have extracellular origin, and the rest are located intracellularly or in the plasma membrane [17]. As for their molecular functions, most proteins have catalytic activity and binding function, followed by receptor and structural functions [16]. About 50% of the follicular proteins are involved in immune activity and coagulation [17]. As indicated above, proteins are the main targets of the attack of reactive oxygen species (ROS), and oxidative stress could compromise their function. In a previous work, we described that controlled ovarian stimulation led to oxidative stress, mainly reflected by changes in the redox status of the serum in terms of total antioxidant activity, susceptibility to in vitro oxidation, and the levels of antioxidants such as tocopherol, bilirubin, uric acid, and albumin [18]. It has been reported that oocytes derived from natural cycles show differences from those from stimulated cycles in relevant aspects, such as epigenetics and ultrastructural integrity [19,20]. Ovarian stimulation with exogenous gonadotropins alters the composition of the follicular milieu [21,22]. However, the effect of the hormonal intervention on the protein integrity is unknown. The aim of this work was to analyze biomarkers of in vivo non-enzymatic oxidative modifications of proteins from human follicular fluid arising from specific pathways, and the impact of ovarian stimulation on these biomarkers. The measured protein oxidation indices were GSA and AASA, which are specific protein carbonyls from metal-catalyzed oxidations, CEL for glycoxidation, and CML for mixed glyco- and lipoxidation.

## 2. Materials and Methods

### 2.1. Study Design and Study Population

Twenty-seven fertile women (with at least one living child) were recruited from 28 February 2014 to 16 December 2015 in the “Oocyte donor program” of the Valencian Institute of Infertility in Bilbao (IVIRMA, www.ivi.es, Vizcaya, Spain). Women were under the age of 35, had a normal physical examination, body mass index between 18 kg/m^2^ and 25 kg/m^2^, and normal karyotype. Criteria for participation in the study were no history of chromosomal or genetic diseases, negative infectious disease screening, regular menstrual cycles (26–35 days), and no history of endometriosis or recurrent miscarriages.

Women underwent both a cycle of controlled ovarian hyperstimulation and a natural cycle. For the stimulated cycles, pituitary desensitization was achieved by administration of GnRH antagonists, as described previously [23]. Briefly, women were given pretreatment with a contraceptive pill (3 mg of drospirenone plus 0.03 mg of ethinyl estradiol (Yasmin; Schering, Madrid, Spain) during the cycle before the scheduled oocytes retrieval. Ovarian stimulation was initiated 5 days after pill discontinuation. A daily dose of 0.25 mg of GnRH antagonist (Cetrotide, Serono Laboratories, Madrid, Spain) was administered on day 6 of stimulation, or when the largest follicle was at least 14 mm in diameter. GnRH antagonist administration was maintained until the induction of ovulation. Ovarian stimulation with gonadotropins (recombinant follicle-stimulating hormone, highly purified human menopausal gonadotropin, or a combination of both) was established for an average of nine or ten days. Ovarian response was assessed by vaginal ultrasound examination every 2 days. Ovulation was induced by single subcutaneous administration of a GnRH agonist (2 mg triptorelin acetate, Gonapeptyl, Ferring S.A., Madrid, Spain) when at least three leading follicles were >17 mm in diameter. Transvaginal oocyte retrieval was scheduled 36 h later.

For the natural cycle, follicle size was assessed by vaginal ultrasound on day 8–10 of the cycle. Thereafter, follicular development was followed every 2 or 3 days until the follicle was between 17 and 20 mm in diameter. Then, ovulation was induced by single subcutaneous administration of triptorelin acetate, and oocytes were retrieved 36 h later.

### 2.2. Ethical Approval

The Ethics Committee of the University UPV/EHU (Ethics Committee for Research involving Human Subjects, CEISH) approved the human subject protocol (CEISH/276/2014/RUIZLARREA), and the study was performed according to the UPV/EHU and IVI-Bilbao agreement. The project complies with the Spanish Law of Assisted Reproductive Technologies (14/2006). Written informed consent was obtained from all trial subjects for participation in the study.

### 2.3. Sample Collection

For each patient, follicular fluid from the sole follicle (natural cycle) or from two contralateral follicles was obtained. Samples visually contaminated with blood were discarded. In the stimulated cycle, samples of follicular fluid from the same woman were pooled. After centrifuging the samples (3000 *g* for 10 min), the supernatant was collected and stored in liquid nitrogen. The samples were taken to the University and kept at −80 °C until analysis.

### 2.4. Measurement of GSA, AASA, CML, and CEL

The oxidative modification products of proteins were determined by gas liquid chromatography coupled to mass spectrometry (GC/MS) with selective ion monitoring, following the method described by Pamplona et al. [24] adapted to follicular fluid. Two milliliters of chloroform:methanol (2:1 *v*/*v*, 3×) in 0.01% butylated hydroxytoluene were added to 20 µl of follicular fluid. Trichloroacetic acid (10% final concentration) was added to precipitate proteins by centrifugation. The proteins were reduced overnight with 1 ml of 500 mM NaBH_4_ (final concentration) in 0.2 M borate buffer, pH 9.2, containing 1 drop of hexanol as an anti-foaming agent. The proteins were precipitated by adding 1 ml of 20% trichloroacetic acid and the solution was centrifuged. The following isotopically labelled internal standards were then added: [^2^H_8_] lysine (*d*8-Lys; CDN Isotopes), [^2^H_4_]-CML, [^2^H_4_]-CEL, [^2^H_5_]-5-hydroxy-2-aminovaleric acid (HAVA) for GSA quantification, and [^2^H_4_]-6-hydroxy-2-aminocaproic acid (HACA) for AASA quantification. The samples were hydrolyzed at 155 °C for 30 min in 1 ml of 6 N HCl, and then vacuum dried. The *N*,*O*-trifluoroacetyl methyl ester derivatives of the hydrolyzed proteins were prepared by sequential treatment with methanolic HCl and trifluoroacetic anhydride [20]. Briefly, 1 ml of 6.5% acetyl chloride in methanol was added to the sample, and the reaction was maintained at 65 °C for 30 min. After vacuum-drying, one milliliter of trifluoroacetic anhydride was added and the reaction proceeded for 1 h at room temperature. The resultant *N*,*O*-trifluoroacetyl methyl ester derivatives were dried and resuspended in dichloromethane for gas-chromatographic studies. The GC/MS analyses were carried out on an Agilent 6890N gas chromatograph equipped with a HP-5MS capillary column (30 m × 0.25 mm × 0.25 μm) coupled to a 5973 mass selective detector (Agilent Technologies, Barcelona, Spain). The injection port was maintained at 275 °C; the temperature program was set at 110 °C for 5 min, with the temperature then rising by 2 °C/min to 150 °C, then by 5 °C/min to 240 °C, then by 25 °C/min to 300 °C, and finally held at 300 °C for 5 min. Quantification was performed by external standardization using standard curves constructed from mixtures of deuterated and non-deuterated standards. The compounds were detected by selected ion-monitoring (SIM) GC/MS. The ions used were: lysine and *d*8-lysine, *m/z* 180 and 187, respectively; HAVA and *d*5-HAVA (stable derivatives of GSA), *m/z* 280 and 285, respectively; HACA and *d*4-HACA (stable derivatives of AASA), *m/z* 294 and 298, respectively; CML and *d*4-CML, *m/z* 392 and 396, respectively; CEL and *d*4-CEL, *m/z* 379 and 383, respectively. The amounts of products were expressed as the ratio of μmol GSA, AASA, CEL, or CML per mol of lysine.

### 2.5. Glucose Measurement

Intrafollicular glucose was determined by glucose oxidase assay kit (Falcorgent GLU from A. Menarini Diagnostics, Barcelona, Spain).

### 2.6. Statistical Analysis

The statistical package SPSS 24.0 (SPSS Inc., Chicago, IL, USA) was used for data analysis. The population size analysis was performed with G*Power 3.1 software (Düsseldorf, Germany) [25]. Sample size was calculated as a function of the power level (0.8), the pre-specified significance level α (0.05), and the population effect size to be detected with a probability of 0.95 (in this case, medium effect size). Taking these parameters into account, a total sample size of 27 was computed. Data were expressed as mean ± standard error of the mean (SE). Statistical significance for the differences of the means was estimated by parametric Student’s *t*-test and the Wilcoxon test (non-parametric equivalent) for paired data. The Pearson’s correlation test was used to analyze associations between quantitative variables. The threshold for statistical significance was set to *p* < 0.05.

## 3. Results

**Figure 1:**
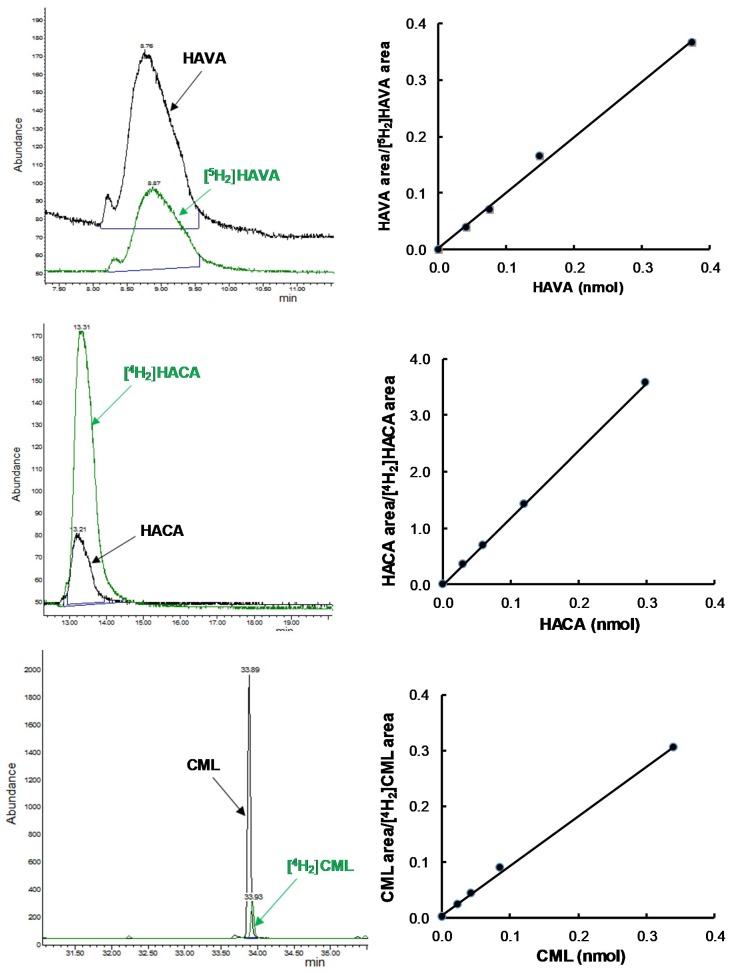

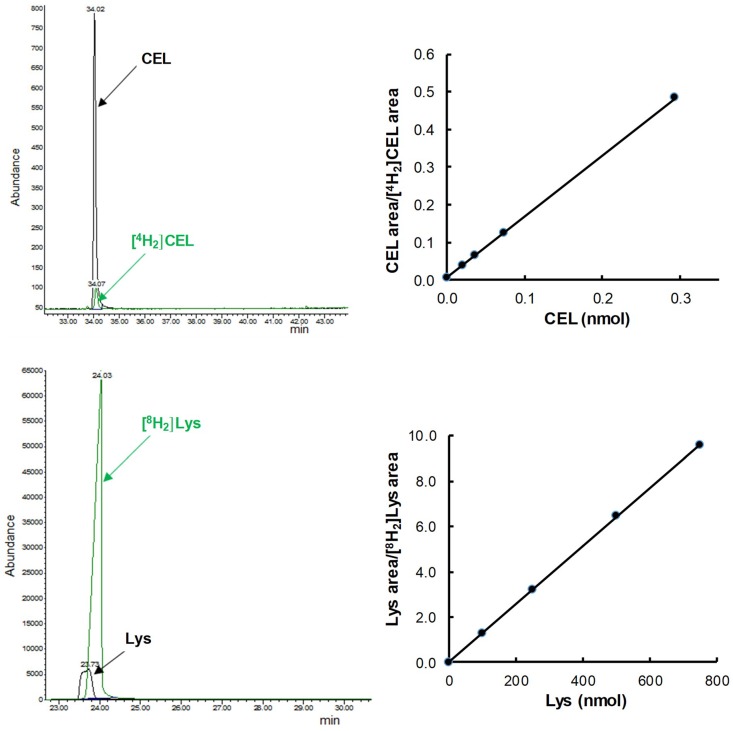
Chromatograms and correlation curves of biomarkers of protein oxidative modifications. Deuterated (green) and non-deuterated (black) compounds were detected in SIM mode. Standard curves with linear correlation coefficients higher than 0.998 were derived from the analyses. HAVA ([^2^H_5_]-5-hydroxy-2-aminovaleric acid) and HACA ([^2^H_4_]-6-hydroxy-2-aminocaproic acid) are the compounds directly detected by GC/MS after sample processing according to Mat and Meth, and correspond to GSA (glutamic semialdehyde) and AASA (aminoadipic semialdehyde), respectively. CML: *N^ε^*-(carboxymethyl)lysine; CEL: *N^ε^*-(carboxyethyl)lysine.

Calibration curves for GSA, AASA, CML, CEL, and lysine were derived in order to have quantitative data by analyzing increasing amounts of the non-deuterated compound in the presence of a constant amount of the corresponding deuterated compound. In the case of direct oxidations by free radicals, HAVA (for GSA quantification) and HACA (for AASA quantification) were used as standards, since during the reduction and acid hydrolysis processing of the samples the parent compounds are converted into HACA (GSA) and HAVA (AASA). Correlation coefficients higher than 0.998 were obtained for all the analyzed protein oxidation indices (Figure 1).

**Figure 2:**
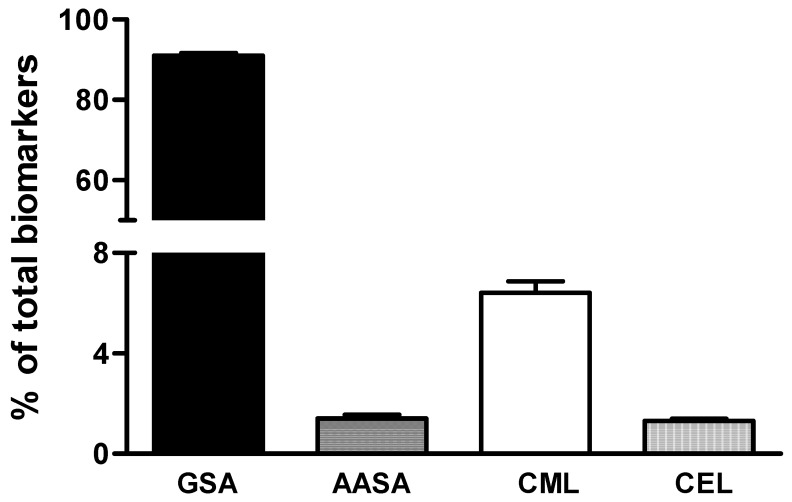
Biomarkers of protein oxidative modifications in follicular fluid. Values were measured in samples from a natural cycle. Bars represent the mean + SE of the percentage of total protein oxidative modifications.

Proteins of follicular fluid contained oxidation products resulting from metal-catalyzed oxidation, glycoxidation, and lipoxidation, since the four mentioned biomarkers were detected in all the analyzed samples (Figure 2). An analysis of their distribution revealed that the most abundant products were those derived from metal-catalyzed oxidation, GSA and AASA (92.8% of the total measured markers). GSA was the most abundant protein lesion (91.5%), corresponding to a mean of 5.1 altered residues per 1000 lysines. The levels of the other analyzed biomarkers were an order of magnitude lower than GSA, reaching between 0.064 and 0.34 damaged residues per 1000 lysines. The levels of CML, a protein oxidation biomarker originated from glycoxidative and lipoxidative processes, amounted to 6% of the total lesions and were higher than the levels of AASA (1.3%) and CEL (1.2%).

**Figure 3:**
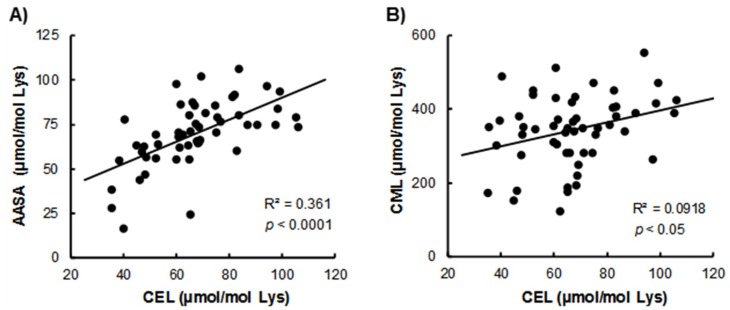
Correlations of (**A**) AASA and (**B**) CML with CEL in follicular fluid. Samples were obtained from natural and stimulated cycles.

Considering all of the measured oxidation indices in natural and stimulated cycles, several correlations between biomarkers were observed (Figure 3). Thus, AASA was significantly correlated with the CEL index of glycoxidative modifications of proteins (*p* < 0.0001). The CML biomarker also correlated with CEL (*p* < 0.05).

**Figure 4:**
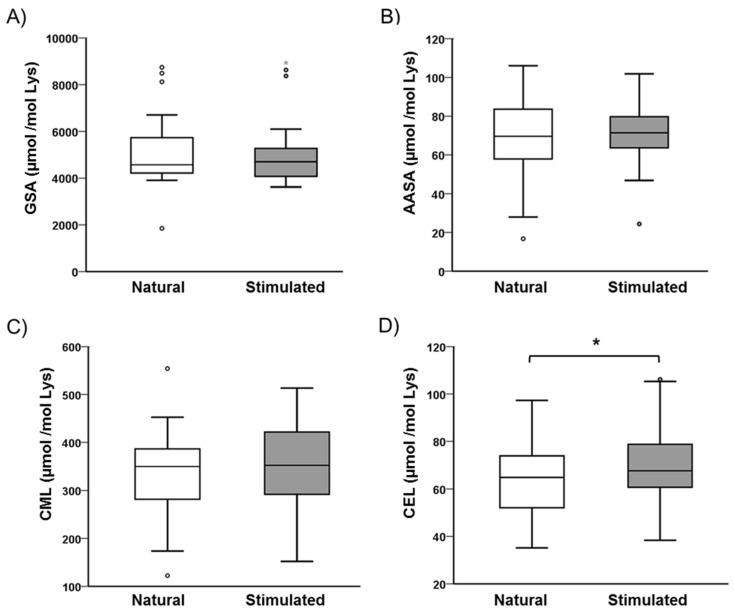
Biomarkers of protein oxidative modifications in follicular fluid from women undergoing both a natural cycle and a stimulated cycle. The box and whiskers graphs represent values for (**A**): GSA; (**B**): AASA; (**C**): CML; and (**D**): CEL. The box extends from the 25th to 75th percentiles. The line in the middle of the box corresponds to the median, and the whiskers are drawn down to the 5th percentile and up to the 95th percentile. Open circles represent the outliers of the distribution. Values are expressed as µmol biomarker/mol Lys residue. * *p* < 0.05.

We used this methodology to determine the possible effect of controlled ovarian hyperstimulation on the protein redox status in the follicular milieu. Results revealed no differences for the metal-dependent protein modifications (Figure 4A,B), nor were the mean values for CML adducts different between the natural cycle (337 ± 18 µmol/mol Lys) and the stimulated cycle (350 ± 19 µmol/mol Lys) (Figure 4C). However, the CEL content in follicular fluid from stimulated cycles was significantly higher than that from natural cycles (8%, *p* < 0.05, Figure 4D).

**Figure 5:**
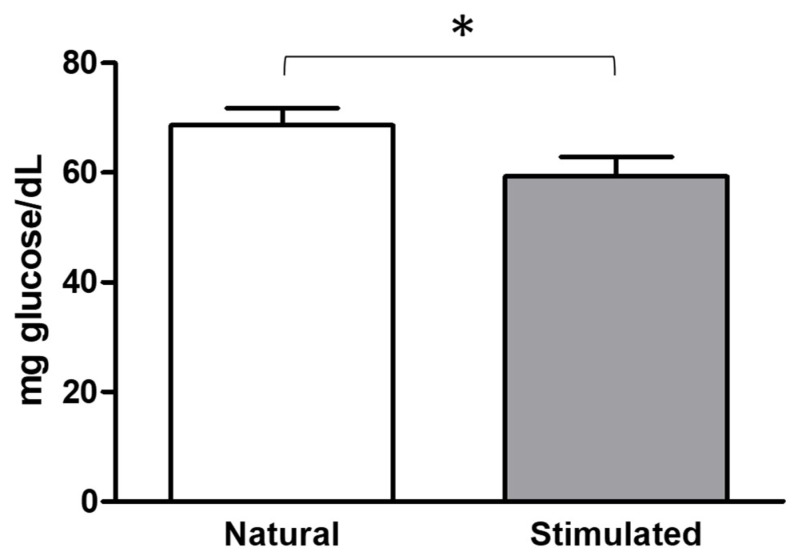
Glucose concentration in follicular fluid from women undergoing both a natural cycle and a stimulated cycle. Bars represent the mean + SE. * *p* < 0.05.

In view of the differences found between natural and stimulated cycles for the index of the modifications of proteins by glycoxidation (Figure 5), we measured glucose in follicular fluid in both cycles. Results showed that the levels of glucose were significantly higher (*p* < 0.05) in the natural cycle (69 ± 4 mg/dl) than in the stimulated cycle (59 ± 3 mg/dl).

## 4. Discussion

In this study, we have described the detection and quantification of specific biomarkers of non-enzymatic oxidative modifications of follicular fluid proteins that arise by different pathways. ROS are products of the cellular metabolic activity that at physiological concentrations play important roles at local and systemic levels. However, non-neutralized ROS cause oxidative damage to lipids, proteins, and nucleic acids, leading to aberrant molecular activities [26]. Oxidative damage to proteins by ROS can result in cleavage of the polypeptide backbone, cross-linking, and modifications of the side chains of amino acids. Various types of protein oxidative modifications are induced directly by ROS or indirectly by reactions with secondary products of oxidative stress. Variations in the steady-state levels of oxidatively modified proteins in vivo can be due to differences in the rates of oxidant generation, antioxidative defenses, protein repair and degradative capacity, or susceptibility of proteins to oxidative damage [2,27]. Our data support the presence of markers derived from protein oxidation in follicular fluid samples from both natural and stimulated cycles determined by gas-liquid chromatography-mass spectrometric. Other authors have reported the presence AGE-modified proteins in follicular fluid, but the analyses were based on immunological techniques [28,29]. The follicular fluid composition reflects metabolic processes and the hormonal microenvironment in which the oocyte develops [30]. Besides granulosa cells, leukocytes and macrophages can also be found in the follicular microenvironment. All of these cell types are capable of generating ROS [31]. The expression of antioxidant enzymes in cells that synthesize steroids, such as granulosa, thecal, and luteal cells, has also been reported [32,33]. As a consequence, the oocyte is exposed in vivo to oxidative stress and ROS scavenging activities present in the follicular fluid.

In the present study, it was clearly shown that the steady-state content of protein carbonyls originated from metal-catalyzed oxidation (GSA and AASA) was higher than the levels of the CEL and CML glycoxidation products. Moreover, the intrafollicular amount of GSA, a product of the oxidation of arginine and proline [6], was higher than that of AASA (which originates from lysine). Taken together, these results indicate that the protein residue oxidation is highly selective and suggest that the degree of oxidation reflected by these biomarkers depends on the amino acid composition, their susceptibility to oxidation (directly affected by the structural characteristics and folding of the protein), and/or the pro-oxidant stimulus. In line with our results, Temple et al. reported that iron-induced in vitro oxidation of human albumin led to the conversion of only two of the 59 lysine residues to AASA, and when hypochlorous acid was used as oxidizing agent five different lysine modification sites were identified [34].

The increase in protein carbonylation may result not only from direct oxidation of the macromolecule by free radicals but also by the involvement of reactive dicarbonyl compounds such as glyoxal and methylglyoxal. These compounds originate during the triose phosphate metabolism, by lipid peroxidation and by myeloperoxidase-dependent reactions [35,36]. Electrophilic aldehydes are well-known by-products of the non-enzymatic peroxidation of polyunsaturated fatty acids, usually arachidonic and linoleic acids, and are also formed from glycolytic intermediates. These aldehydes are highly reactive with protein residues of histidine, cysteine, and lysine, forming Michael’s adducts, which may result in impairment of enzyme activities and interference with signaling pathways [37,38,39]. Amino acid residues, such as lysine, can be modified by adduct formation with aldehydes derived from the peroxidation of polyunsaturated fatty acids, particularly aldehydes with α, β-unsaturated functional groups [40]. Among them, glyoxal, a precursor to CML, yields chemically stable adducts with lysine residues. CML was initially described as a product of carbohydrate autoxidation, but it is now well documented that it is also a compound derived from lipid peroxidation in the presence of proteins, and its amount depends on the degree of unsaturation and oxidizability of fatty acids [41]. We have detected CML in the follicular fluid of women at a concentration about 10-fold lower than the metal-dependent modifications.

Neither the CML nor the GSA and AASA levels in the follicular fluid were modified as a consequence of controlled ovarian stimulation. By contrast, we observed a higher content of CEL in follicular fluid from stimulated cycles. We do not know the physiological relevance of this 8% increase, although a similar increase of CEL has been reported in brain cortex samples from patients with Alzheimer’s disease [24]. It is still not known whether the damaged proteins associated with this marker have specific functions in the reproductive process. AGEs in the follicular fluid not only represent a loss of function of those AGE-modified proteins but are also an additional source of ROS. AGEs circulate in the medium (i.e., follicular fluid) and bind to plasma membrane receptors, such as the receptor for AGEs (RAGE), which is a member of the immunoglobulin superfamily of receptors [42]. The AGE/RAGE interaction activates the nuclear factor kappa-B and other downstream pathways, resulting in production of ROS and pro-diabetic, pro-inflammatory, pro-thrombotic, and pro-atherogenic responses [8]. In a recent study, a proteome analysis of follicular fluid from natural and stimulated cycles revealed that eight proteins were differentially expressed in stimulated cycles [43]. The upregulated proteins were related to immune and inflammatory responses, suggesting that these processes might play a role in the adverse effects of ovarian hyperstimulation. The treatment with exogenous gonadotropins leading to multiple oocyte retrieval would contribute to ROS increase, and consequently their detrimental effects on proteins.

The CEL increase in the stimulated cycle was accompanied by a substantial reduction in follicular glucose levels. This decrease could be due to the fact that the sugar is being metabolized to intermediates that generate methylglyoxal, which reacts with intrafollicular proteins, thus forming stable adducts. The ovarian stimulation protocol has been shown to alter the intrafollicular levels of hormones associated with fertility parameters, in particular estradiol, luteinizing hormone, and anti-Müllerian hormone. The treatment with exogenous gonadotropins reduced these hormones, reflecting poorer outcomes of oocyte maturation and quality, and embryo implantation [21]. It is unknown whether the hormonal alterations are related to the changes observed in CEL levels or if they are independent factors that may contribute to the in vitro fertilization (IVF) outcomes.

The present work is a pilot study. If the results are validated using a larger sample size, the measurement of CEL could have implications in assisted reproduction. The identification of the specific glycoxidatively modified proteins and the knowledge of the associated functional changes in fertility parameters are a subject of research to develop. The information derived from those data could result in direct implications for the techniques and outcomes of assisted reproduction. It also remains to be established whether similar changes are reflected in other biological fluids, such as blood.

## 5. Conclusions

The GC/MS method described in this article provides a means for the specific quantification of CEL and CML adducts and oxidative modifications of amino acids (GSA and AASA) occurring in follicular fluid proteins. Differences between the natural and stimulated cycles were found for the CEL glycoxidation-modified protein biomarker, with the lowest index observed for the natural cycle. These findings are the basis for future research and open new perspectives for establishing the possible relevance that this intrafollicular biomarker of in vivo protein modifications has in determining success of IVF in assisted reproduction programs.
